# Improving Long-Term Adherence to Endocrine Therapy Among Breast Cancer Survivors: Development of a Multiscale Modeling and Intervention System

**DOI:** 10.2196/68255

**Published:** 2026-04-30

**Authors:** Manuel Gonzales IV, Cristian Garcia-Alcaraz, Navreet Kaur, Anna N Baglione, Sarah Livermon, Laura E Barnes, Kristen J Wells

**Affiliations:** 1 SDSU/UCSD Joint Doctoral Program in Clinical Psychology, 5500 Campanile Drive, San Diego, CA, 92182, United States, 1 619-594-1919; 2 Department of Engineering Systems and Environment, University of Virginia, Charlottesville, VA, United States; 3 Department of Psychology, San Diego State University, San Diego, CA, United States

**Keywords:** breast cancer survivors, social cognitive theory, mobile app, mHealth, endocrine therapy, medication adherence, breast neoplasms, maintenance chemotherapy, ecological momentary assessment, digital health

## Abstract

**Background:**

Breast cancer is a significant public health burden. Despite its critical role in preventing the recurrence of breast cancer, rates of long-term adherence to endocrine therapy (ET) remain low among certain breast cancer survivors. Using embedded sensors in smartphones and wearables, ecological momentary assessment data and health behavior theory may facilitate a richer understanding of the real-world context of medication-taking behaviors, which can aid in the development of personalized interventions.

**Objective:**

The objective of this paper is to describe the development of a multiscale modeling intervention (MMI) system to facilitate adherence to daily oral ET for breast cancer survivors. This represents the first phase of a larger project that aims to use machine learning to predict when breast cancer survivors are most likely to miss their ET medications in order to deploy personalized interventions. The purpose of this paper was (1) to determine the acceptability of the proposed MMI system, (2) to identify modifiable predictors of ET medication adherence among breast cancer survivors, and (3) to select surveys or items measuring constructs associated with ET adherence among breast cancer survivors for inclusion in the MMI system.

**Methods:**

Study 1 consisted of usability interviews with a cohort of breast cancer survivors (n=25) prescribed ET. For study 1, all qualitative usability interviews were conducted using a semistructured interview guide and assessed whether breast cancer survivors were willing to use various components of the MMI system. Study 2 consisted of (1) a secondary data analysis of ET adherence data from 32 breast cancer survivors using a social cognitive theory framework and (2) a review of research literature of constructs and surveys measuring constructs associated with ET adherence among breast cancer survivors using a social cognitive theory framework. The secondary data analysis included the use of randomized neural network analysis to predict factors strongly associated with medication adherence.

**Results:**

In study 1, usability interview findings suggested that participants were willing to use an ecological momentary assessment smartphone app, a smartwatch and associated smartphone app, a smart pill bottle or smart pill box and associated smartphone app, and the entire MMI system for a 6-month study period. In study 2, the randomized neural network analysis identified 104 survey items with significant contributions to 4-week medication adherence using a threshold of the 70th percentile for feature importance. After a review of peer-reviewed studies, we abstracted modifiable constructs significantly associated with adherence to adjuvant ET and identified 42 surveys used to measure these constructs. When these findings were combined, the final survey for the MMI system consisted of 32 surveys and demographic items.

**Conclusions:**

Our research highlights the use of social cognitive theory, data-driven models, and participant feedback to inform the development of a medication adherence monitoring system. Data from studies 1 and 2 were used to develop a prototype MMI system that will be deployed in a future longitudinal study with 20 breast cancer survivors over 6 months.

## Introduction

Breast cancer is one of the most commonly diagnosed cancers worldwide [1]. In 2025, an estimated 319,750 people will be diagnosed with breast cancer, and 42,680 people will die from it in the United States [2]. Additionally, there are approximately 4.1 million breast cancer survivors in the United States [3]. Based on the most recent statistics, in the United States, the 5-year relative survival rate for breast cancer from the years 2014 to 2020 was 91% [2]. Individuals diagnosed with early stage and nonmetastatic breast cancer typically undergo a treatment regimen that can include surgery (ie, lumpectomy and mastectomy), radiation, chemotherapy (including trastuzumab), endocrine therapy (ET), immunotherapy, or a combination of treatments. The selection of treatment approaches is based on the stage of the cancer, whether breast cancer cells have certain receptors, and a patient’s overall health and age [4-6]. Treatment for an early stage of breast cancer aims to eliminate the cancer, prevent recurrence, and maximize quality of life.

Giaquinto et al [3] reported that 80% of breast cancers diagnosed from the years 2015 to 2019 were estrogen receptor–positive. Survivorship guidelines developed by the American Cancer Society and American Society of Clinical Oncology recommend that breast cancer survivors with estrogen receptor–positive breast cancer take daily oral ET, including tamoxifen or aromatase inhibitors, for 5 to 10 years following treatment with surgery, chemotherapy, and radiation to reduce the risk of recurrence of breast cancer and subsequent secondary primary breast cancers [7]. ET consists of 1 pill taken at any time of the day, 1 time per day, with or without food. Individuals who take exemestane are required to avoid grapefruit and Seville, that is “bitter” oranges, as they reduce its efficacy [8]. Additionally, individuals taking ET should avoid or limit alcohol, as it reduces the efficacy of the medications [8].

Breast cancer survivors who take tamoxifen for 5 years posttreatment have a one-third reduced risk of breast cancer mortality through 15 years after diagnosis and an average of 39% reduced recurrence rate through 10 years after diagnosis compared to those who are not using tamoxifen [9]. Van Liew et al [10] conducted a systematic review of adherence to oral ET and found that breast cancer survivors who adhered to ET for more than 80% of the time had the lowest risk of the recurrence of breast cancer. Rates of long-term adherence to ET are low among certain breast cancer survivors despite its critical role in preventing the recurrence of breast cancer. Estimated rates of adherence to ET range from an average of 79% during the first year after treatment to an average of 56% during the fourth or fifth year [11]. A recent systematic review examining modifiable factors associated with adherence to ET identified the presence of side effects as being associated with ET adherence (in 53% of univariate studies and 100% of multivariate studies) [12]. Furthermore, positive decisional balance, positive emotions, negative emotions, depressive symptoms, perceived risk of breast cancer recurrence, intention to take ET, self-efficacy, participation in the decision to take ET, and quality of the relationship with a health care provider were shown to be associated with ET adherence across multiple studies. A recent systematic review and meta-analysis found that, overall, interventions that aimed to improve adherence to ET were effective, but there was considerable variation across the studies, with 53.3% of randomized controlled trials and 57.9% of nonrandomized controlled trials reporting an intervention effect to improve adherence. Interventions that reduced the cost of medications through legislation and the use of generic medications were consistently effective. Psychosocial interventions that focused on individual participants had limited efficacy when they were provided education about breast cancer, ET, and side effects. Those psychosocial interventions that targeted forgetting and involved medical personnel in managing side effects were sometimes effective [13]. Some psychosocial interventions to improve ET adherence may have limited effectiveness because there is a poor understanding of barriers to adherence and circumstances in which medication taking occurs or does not occur.

Further examination of adherence to ET may be crucial in reducing the burden of breast cancer and improving the overall quality of life and long-term survival among breast cancer survivors. The collection of ecologically valid data, such as sensors (ie, wearable sensors, medication event monitoring system [MEMS] pill cap sensors, and smartphone sensors) and ecological momentary assessment (EMA) data, may facilitate a better understanding of medication-taking behaviors. Baglione et al [14] described the framework of a multiscale modeling intervention (MMI) that presents a new approach to intervention development by capturing real-time data using mobile and wearable sensing systems and working toward incorporating theory-driven approaches to intervention development for adherence to ET. Through the lens of social cognitive theory (SCT) [15], identifying personal (ie, physiological, cognitive, and affective states), environmental (ie, social and physical environment), and behavioral (ie, medication-taking in the context of other behaviors) influences that impact adherence to ET may help provide better insights into the context of adherence and nonadherence. The combination of a theory-driven approach along with data collection using smartphone and wearable sensors, surveys, and EMA will allow for a much richer understanding of the real-world context of medication-taking behaviors, which will ultimately aid in the development of personalized interventions.

As part of the current project, in 2020, the study team conducted an informal assessment of commercially available wearable and MEMS sensors that could be used to collect ecologically valid data from breast cancer survivors to better understand their risk of ET nonadherence and aid in the development of an MMI system. Wearable sensors and MEMS devices were identified via online searches and through the knowledge of study team members. Sensors were reviewed and tested based on a wide variety of factors that would be important for feasible data collection and intervention as part of the MMI system (eg, cost, battery life, platform, etc; see Tables S1 and S2 in Multimedia Appendix 1). All data obtained from the review and testing of the MEMS devices were entered into an Excel spreadsheet. Multiple members of the project team reviewed these data to make final sensor selections. Based on this review, the team selected Fitbit Sense as the final wrist-worn sensor and RxCap as the final MEMS device for the MMI system.

**Figure 1.**
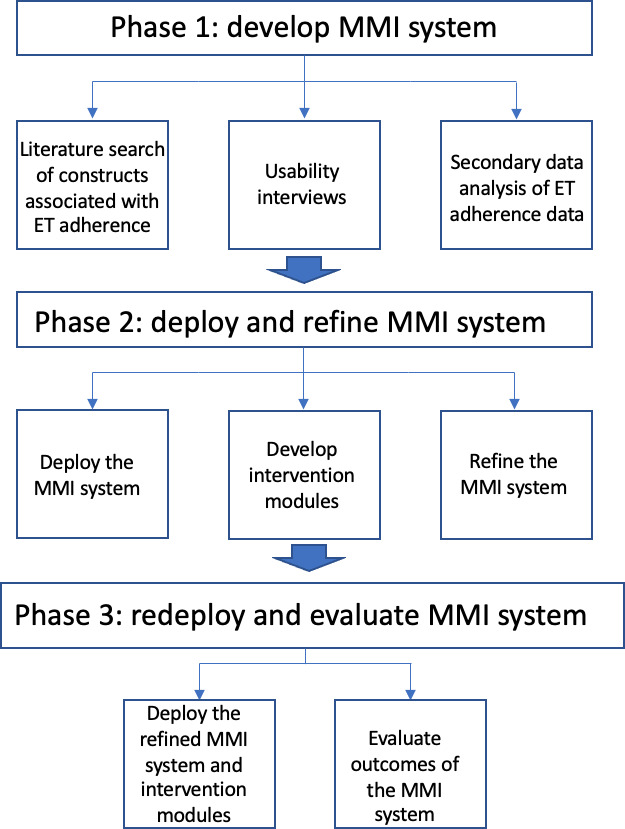
Flow diagram of multiscale modeling intervention (MMI) for medication adherence project. ET: endocrine therapy.

In this paper, we describe the development of an MMI system to facilitate adherence to daily oral ET for breast cancer survivors who were diagnosed with stages 0 to 3 breast cancer in the past 5 years, have completed all surgeries, radiation, and chemotherapy treatments for breast cancer, and were prescribed ET. This represents the first phase of a larger project (Figure 1) that aims to use machine learning to predict when breast cancer survivors are most likely to miss their ET medications to deploy personalized interventions. The purpose of this initial phase was (1) to determine the acceptability of the proposed MMI system, (2) to identify modifiable predictors of ET medication adherence among breast cancer survivors, and (3) to select surveys/items measuring constructs associated with ET adherence among breast cancer survivors for inclusion in the MMI system. To develop the MMI system, we conducted 2 studies. Study 1 consisted of usability interviews with 25 breast cancer survivors prescribed ET. Study 2 consisted of (1) a secondary data analysis of ET adherence data from 32 breast cancer survivors and (2) a review of research literature of constructs and measurement of constructs associated with ET adherence among breast cancer survivors. The information obtained from these 2 studies allowed us to (1) develop long-form and EMA surveys and determine the frequency of administration of EMAs and (2) determine survey items and sensor data critical to include in the deployment of the prototype MMI system.

## Methods

### Overview

This phase of the project describes the development of the MMI system for a future 6-month deployment of the system among 20 breast cancer survivors.

### Study 1: Usability Interviews With Breast Cancer Survivors

#### Participants

Using purposive sampling [16], participants were breast cancer survivors (n=25 women) prescribed ET who were recruited via social media posts (eg, Facebook) targeted toward patients diagnosed with breast cancer. These posts were made in breast cancer–related groups and channels. Those who were interested were asked to respond to the post with their contact information. A team member subsequently communicated with individuals who expressed interest in participating via email. Purposive sampling, unlike random sampling, is used to select cases who will provide appropriate and useful information [16]. Participants who expressed interest in participating in the study were sent an eligibility screener on Qualtrics. Eligibility criteria included participants who (1) had the ability to speak and read English, (2) were between the ages of 21 and 70 years, (3) were diagnosed with stages 0 to 3 breast cancer in the past 5 years, (4) had completed all surgeries, radiation, and chemotherapy for breast cancer, and (5) were prescribed ET. If a participant was deemed eligible based on the Qualtrics screener, they were subsequently contacted and scheduled for a screening via Zoom videoconference in order to confirm eligibility. If eligibility was confirmed, participants were scheduled for a 60- to 90-minute semistructured interview via Zoom videoconference. Interviews took place between September 2020 and July 2022.

#### Procedure

Using semistructured interview guides, 2 doctoral-level researchers (MG and CG-A) conducted 5 rounds of interviews assessing breast cancer survivors’ perceptions of and willingness to use an EMA smartphone app, smartwatch, smart pill bottle, smart pill box, and the entire MMI system. All interview guides were developed by the study team and pilot-tested with undergraduate research assistants and individuals outside of the study team (see Multimedia Appendix 2 for all interview guides). One interviewer (MG), a male doctoral student, held a bachelor’s degree in psychology, significant interest in systematic approaches to intervention development, and prior experience in clinical health psychology research. The second interviewer (CG-A), a male doctoral student, held a master’s degree in psychology, significant interest in research methodology, and prior experience in qualitative research. Each round consisted of 5 participants who were not known to the researcher conducting interviews, and only 1 interviewer was present during each semistructured interview. Five interviews were conducted in each round based on the experience with prior human-centered technology design research [17]. Participants were interviewed alone. There were no repeat interviews. The purpose of round 1 interviews (n=5 participants) was to present a short survey on an EMA smartphone app and ask about participants’ willingness to use and their thoughts on the usability of the EMA application. The purpose of round 2 (n=5 participants) was to present a smartwatch and EMA smartwatch application and ask about participants’ willingness to use it and their thoughts on the usability of the smartwatch and associated smartphone app. The purpose of rounds 3 (n=5 participants) and 4 (n=5 participants) was to ask about participants’ current medication-taking behaviors, demonstration of the use of a smart pill bottle and box, willingness to use a smart pill bottle or smart pill box, and willingness to use a smartphone app associated with the smart pill bottle. The purpose of round 5 (n=5 participants) was to ask about participants’ willingness to use the entire MMI system, including completion of the long-form and EMA surveys, demonstration of the use of a pill bottle sensor, using a smartwatch, using apps associated with the 2 sensors, and their comfort with the EMA application collecting various streams of data (eg, HealthKit, activity, and Bluetooth encounters). All interviews were audio-recorded using a digital audio recorder. In addition, participants completed a short survey using Qualtrics, which assessed the following sociodemographic and clinical variables: age, Latinx ethnicity, race, education, marital status, sex assigned at birth, gender identity, employment status, occupation, country of birth, years residing in the United States, primary language, number of people in the household, annual household income, year of breast cancer diagnosis, stage of breast cancer diagnosis, types of treatment for breast cancer, and whether they experienced a breast cancer recurrence.

#### Data Analysis

All recorded audio files were transcribed verbatim by 2 undergraduate research assistants. Transcripts were not returned to participants for comment. A member of the research team (MG) reviewed all transcripts and developed a summary document for each of the 5 rounds of usability interviews (n=5 for each round). Data were summarized based on the participants’ answers to specific questions in the interview, such as “*In general, how do you feel about the experience of answering the surveys on the smartwatch?”* For some items, responses were summed (eg, how many participants stated it was easy to use a feature). Descriptive statistics (ie, frequencies, means, and SDs) of participant characteristics were calculated via SPSS (version 29; IBM).

### Ethical Considerations

This cross-sectional qualitative study was approved by the University of Virginia Institutional Review Board (IRB) (IRB-HSR number 21406) to which the San Diego State University and University of Alabama IRBs agreed to rely on the regulation of the study. This paper follows COREQ (Consolidated Criteria for Reporting Qualitative Research) reporting guidelines for qualitative research (a complete COREQ checklist can be found in Checklist 1) [18]. All participants provided written consent prior to starting the usability interviews, which took place between September 2020 and July 2022. Data were stored in accordance with the University of Virginia’s information security policies, and deidentified data were analyzed. Participants were provided with a US $75 gift card for completion of the 60- to 90-minute semistructured interview and self-report survey. This study adhered to local, national, regional, and international law and regulations regarding protection of personal information, privacy, and human rights.

### Study 2

#### Secondary Data Analysis

As previously reported by study team members Kaur et al [19], to select long-form surveys and EMA items/surveys, the research team analyzed data from 32 early-stage breast cancer survivors [19,20]. In the primary study from which these data were obtained, eligible participants provided informed consent after participating in the screening process. After completing baseline surveys during the primary study, breast cancer survivors’ daily medication adherence was assessed using a MEMS pill bottle (Aardex) over the 8-month time period. As part of the primary study, participants were compensated with gift cards worth up to US $90: US $30 for the baseline survey, US $30 for the 4-month survey, and US $30 for the 8-month survey. The data from the primary study were stored in accordance with San Diego State University and Sharp HealthCare Information Security policies, and deidentified data were analyzed by Kaur et al [19]. The initial IRB approval by the San Diego State University IRB (vIRB number 1239088) and the Sharp HealthCare IRB (IRB number 1308810) permitted the secondary use of data.

Kaur et al [19] conducted a randomized neural network (RNN) analysis to determine important features (eg, attitudes toward medications, stress, and mood) of ET adherence 4 weeks following the completion of the 346-item baseline survey. Kaur et al [19] extracted the weights on different input variables of the trained RNN models, which are equivalent to the contribution of the respective variables in the final outcome prediction. Additionally, Kaur et al [19] were guided by principles of SCT [15], which evaluates how environmental factors, personal factors, and a person’s behavior interact through a process of reciprocal determinism. In other words, the SCT indicates that each of these aspects (eg, environmental factors, personal factors, and person’s behavior) influences each other dynamically, which is important for the MMI system. Therefore, Kaur et al [19] used the SCT [15] when identifying personal, behavioral, and environmental constructs associated with daily medication adherence in the RNN analysis. Two authors (KJW and LEB) were largely responsible for categorizing constructs as personal, behavioral, or environmental factors associated with daily medication adherence in the RNN analysis. These findings were presented to other members of the research team for their feedback, which were incorporated by a third author (NK). Details regarding the RNN analyses are reported in the prior publication [19]. Kaur et al [19] chose the survey item importance starting with the fourth week of ET MEMS data collection, as the Hawthorne effect associated with the new use of the MEMS cap and enrollment in the study would have attenuated by week 4. In contrast, during weeks 2 and 3, participants were probably still affected by the study enrollment, completing the baseline surveys, and acclimating to the MEMS cap. Thus, only the 4-month and 8-month data were used to prevent noise from baseline data from corrupting the models. Kaur et al [19] used descriptive statistics to summarize demographic and clinical data. Details of the final survey items are provided in the *Results* section.

#### Review of Research Literature

Because the secondary analysis of data did not include all potential modifiable factors associated with ET medication adherence, we also examined the systematic review of Toivonen et al [12] (2020) to further develop the long-form survey and EMA components for the MMI system. The synthesis of Toivonen et al [12] comprised 68 studies that examined modifiable factors associated with adherence to adjuvant ET in breast cancer survivors. Possible modifiable factors of adjuvant ET in the literature were classified into the following 6 categories by Toivonen et al (2020) [12]: side effects (n=44 studies), attitudes toward adjuvant ET (n=29 studies), psychological factors (n=30 studies), health care provider–related factors (n=26 studies), sociocultural factors (n=25 studies), and general/quality-of-life factors (n=24 studies). Across individual studies within the synthesis of Toivonen et al [12], 1 study team member and author (CG-A) abstracted which modifiable factors were significantly associated with adherence to adjuvant ET as well as the different surveys/items used to measure these factors. One author (CG-A) also retrieved articles describing the development and psychometric evaluation of such surveys or items. This author (CG-A) then collaborated with another author (KJW), who has expertise in survey development, to determine the quality of these surveys or items. Surveys or items were considered for initial inclusion in the long-form survey and EMA components for the MMI system if they had adequate psychometric properties based on a minimum of a Cronbach *α*≥0.70 in at least 1 study sample (when psychometric information was available).

The final inclusion of items and surveys in the long-form and EMA surveys was determined by the monetary cost, length of the surveys and items, and the previously described secondary data analysis findings. To be included in the MMI system, the surveys/items had to be free or a low cost, short enough to ensure the completion of the full battery of long-form surveys within 45 minutes or completion of EMA surveys within 5 minutes, and measure significant modifiable factors of adherence to adjuvant ET according to the secondary data analysis and the systematic literature review of Toivonen et al [12]. All authors participated in the decision-making process to select items and surveys during weekly team meetings in which the RNN analysis and the literature review were discussed. For the long-form surveys, a draft of the baseline survey was created by one author (CG-A), revised by a second author (KJW), and then presented to all group members for their suggestions and revisions. Once the baseline survey was finalized through consensus of the study team, the 2 follow-up surveys were created by one author (CG-A) and edited by a second author (KJW). The follow-up surveys were then presented to the study team for their suggestions and revisions. The EMA surveys were drafted by 2 study authors (ANB and KJW) and reviewed by all other study team members over several rounds of revisions. The long-form and EMA surveys were finalized once the study team agreed that no further edits were required.

## Results

### Study 1

#### Sample Characteristics

Out of 25 participants, 84% were White (n=21), 4% were African American/Black (n=1), and 12% were identified as Hispanic/Latinx (n=3; Table 1). The mean age of the sample was 53.6 (SD 10.1) years. Twenty-two out of 25 participants were married (88%), 13 were employed for wages (52%), 22 of them were born in the United States (88%), 24 had a primary language of English (96%), 10 were diagnosed with stage 1 breast cancer (43%), and 24 of them did not have a breast cancer recurrence (96%). Twenty-nine respondents agreed to participate in the interviews and 25 were interviewed (86% completion rate). Three participants who were scheduled to participate in an interview did not attend the appointment (75%), and 1 indicated she was no longer interested in participating prior to the interview (25%).

**Table 1.** Sample demographics and clinical characteristics of study 1 participants.

Characteristic and variable	Value
Race/ethnicity (n=25), n (%)	
White	21 (84)
African American/Black	1 (4)
Hispanic/Latinx	3 (12)
Sex (n=24), n (%)	
Female	24 (100)
Gender (n=25), n (%)	
Female	25 (100)
Marital status (n=25), n (%)	
Legally married	22 (88)
In a committed relationship	1 (4)
Divorced/separated	1 (4)
Widowed	1 (4)
Employment status (n=25), n (%)	
Employed for wages	13 (52)
Self-employed	2 (8)
Out of work	1 (4)
Homemaker	3 (12)
Student	1 (4)
Retired	5 (20)
Country of birth (n=25), n (%)	
United States	22 (88)
Canada	1 (4)
Denmark	1 (4)
Ukraine	1 (4)
Annual household income in US dollars (n=25), n (%)	
10,000-19,999	1 (4)
20,000-29,999	0
30,000-39,999	0
40,000-49,999	0
50,000-74,999	0
75,000-99,999	8 (32)
100,000-124,999	5 (20)
125,000-149,999	2 (8)
150,000 or more	9 (36)
Primary language (n=25), n (%)	
English	24 (96)
Danish	1 (4)
Breast cancer stage (n=23), n (%)	
Stage 0	2 (9)
Stage 1	10 (43)
Stage 2	5 (22)
Stage 3	6 (26)
Type of treatment (n=25), n (%)	
Single mastectomy	3 (12)
Double mastectomy	11 (44)
Lumpectomy	13 (52)
Another type of surgery	1 (0.04)
Radiation	17 (68)
Chemotherapy	16 (64)
Other	4 (16)
Breast cancer recurrence (n=25), n (%)	
Yes	1 (4)
No	24 (96)
Age in years (n=25), mean (SD)	53.6 (10.1)

#### Round 1 Interviews

All participants stated that filling out the survey on the smartphone app was easy and straightforward, and they would find it easy to use the smartphone app on their own phones and are willing to use the app for the future 6-month study duration. Additionally, several participants reported preferences for completing a survey up to 2 times per day with up to 10 questions at a time. Participants also had preferences for completing surveys early in the day or later in the evening. Furthermore, a few participants reported concerns about “being a slave to their phone,” receiving multiple notifications throughout the day while busy, and being busier on the weekends. However, participants reported that they would prefer an option to receive a notification to complete a survey and be able to attend to it at a later time. Finally, participants reported recommendations for lighter colors in the mobile app, the ability to customize the colors of the app, and the inclusion of larger text.

When asked about how easy or difficult it was to answer the questions using the EMA smartphone app, one participant said:

*That was easy...That was easy. I think that the answers were just, it was laid out very simple. There wasn’t a lot of distraction; so the answer was very easy to just select, to read the question and select the appropriate answer.*

[P3]

#### Round 2 Interviews

Participants described the use of the smartwatch as easy, user-friendly, and clear and liked it as it was convenient, easy, and fast. Most participants reported that they would find it easy to remember to wear the watch every day for the duration of the 6-month study period, would accept answering questions on the smartwatch application for the 6-month study period, and that the use of the app on the smartphone would be easy. Several participants also reported that they would not prefer to wear the watch while showering, sleeping, or dressing up in a nice outfit. Several participants preferred larger text on the app and stated that the smartwatch demonstrated was too big. Only 1 participant reported that they might have difficulty using a smartwatch for the 6-month study duration due to not having a preference for wearing jewelry or watches.

When asked about their experience of answering surveys on a smartwatch emulator, one participant said:

*I think it’s very easy, very user friendly. So, I think it’s great...I think, so if that just pops up on your watch without you having to do anything, it’s very user friendly; it has the options there. Anybody could do it—like a 5-year-old could do it.*

[P23]

#### Rounds 3 and 4 Interviews

The mean number of reported medicines taken was 3.1 (SD 1.4), and the mean number of vitamins or supplements taken was about 3.6 (SD 2.1). Almost all participants reported taking their medicines or supplements in the morning and/or in the evening. A majority of participants stored their medicines and supplements in the kitchen, on their nightstand, or a combination of both, and 5 participants reported keeping their medicines in a pill box, while 4 reported keeping them in their original container. Of note, only 2 participants reported having someone who helps them manage their medications. Generally, participants were willing to use the smart pill bottle or smart pill box and the associated smartphone app for the duration of the 6-month study period.

When asked about how easy or difficult it would be to fit using the smart pill bottle into their everyday life, one participant said:

*I don’t think it would be difficult at all...I mean I think it would be something that, let’s say that pharmacy gave to me and said here do you want to try this out, I would, I would absolutely try it.*

[P42]

When asked about how easy or difficult it would be to fit using the smart pill box into their everyday life, one participant said:

*I think it’d be fairly easy to use...It’s, it’s got every day’s medication. I see it goes by the day. It’s going to tell me if I took it. And it’s not that big or bulky...I would say it’s of decent size.*

[P71]

#### Round 5 Interviews

In general, participants were willing to use the entire MMI system for the duration of the 6-month study period. However, participants reported that the survey (see below) they completed was too long; several participants requested that the survey be shortened. In general, participants were comfortable with the EMA app collecting various streams of data and preferred the option to select which streams of data were collected or not collected. However, 2 participants described the collection of data streams as “intrusive.”

When asked about their willingness to use the entire MMI system for 6 months of data collection, one participant said:

*Yeah. I would...Because I think it’s important to find ways to help other people. I think that’s important, but yeah; and I like the fact that I can opt out of certain things.*

[P83]

### Study 2

#### Survey and EMA Evaluation

As described by Kaur et al [19], data from 32 breast cancer survivors who completed surveys at all 3 time periods (baseline, 4 mo, and 8 mo; Table 2) were used in the RNN. Of the 32 breast cancer survivors who participated in the study, 29 (90.6%) had MEMS data for the entire 8-month period. As indicated by Kaur et al [19], the analysis included bottle-opening events before or after the survey periods to evaluate the algorithms’ prediction performance. A total of 2604 data samples were adopted, and each one included subjective values from survey data and the ground truth of medication-taking behavior captured by the MEMS device.

**Table 2.** Sample demographics and clinical characteristics of study 2 participants^a^.

Characteristic and variable	Value
Race (n=32), n (%)	
White	14 (44)
African American/Black	4 (13)
Asian	2 (6)
Native Hawaiian/Other Pacific Islander	1 (3)
Native American/Alaskan Native	1 (3)
Unknown	10 (31)
Ethnicity (n=32), n (%)	
Latin (o/a/e/x)	25 (78)
Not Latin (o/a/e/x)	7 (22)
Gender (n=32), n (%)	
Female	32 (100)
Preferred language (n=32), n (%)	
English	7 (22)
Spanish	24 (75)
Other	1 (3)
Breast cancer stage (n=32), n (%)	
DCIS^b^	5 (16)
Early breast cancer	25 (78)
Locally advanced breast cancer	2 (6)
Intervention arm (n=32), n (%)	
Patient navigation intervention	15 (47)
Usual care	17 (53)
Age in years (n=25), mean (SD)	51.6 (8.9)

a Adapted from Kaur et al [19].

b DCIS: ductal carcinoma in situ.

As described by Kaur et al [19], the developed RNN models outperformed traditional neural network models in terms of adherence prediction accuracy under 2 types of randomness: subjective values and decision rules randomness. After training the adherence prediction models, we preferred to gain insights into the importance of different input variables toward medication adherence prediction. We extracted the weights on different input variables of the trained RNN models, which are equivalent to the contribution of the respective variables in the final outcome prediction. We had 346 individual items as input variables, and using a threshold of the 70th percentile for feature importance (absolute input variable weights), we ended up with 104 survey items with significant contributions to 4-week medication adherence. Within the context of SCT [15], RNN analysis findings identified the following constructs associated with daily medication adherence: (1) environment: patient-provider interactions; instrumental, emotional, and informational social support; barriers to health care; and patient satisfaction with care; (2) personal: perceptions of control/agency/self-efficacy, numerous medication-related side effects, spirituality, perceived stress, decision regret, and fatigue; and (3) behavioral: having a routine for medication taking.

With regard to systematic review findings [12], 42 surveys were reviewed as predictors of ET medication adherence. In addition, there were additional SCT [15] constructs found to be associated with ET medication adherence among breast cancer survivors, as indicated by a recent systematic review [12]: (1) environment: communication with a health care provider and health care provider relationship quality, and (2) personal: mood/depression, perceived cognitive function, and perceived susceptibility of recurrence.

When combined, the RNN analysis [19] and the review of existing literature resulted in the identification of 34 surveys that could be included in the MMI system during the planned deployment, measuring constructs identified in both approaches as important predictors of ET adherence. Given participant feedback on reducing the length of the survey to be administered (see Round 5 usability interview findings), 2 measures of cancer health-related quality of life were removed due to the lengthiness of the surveys and because it was believed that health-related quality of life was also assessed by other measures being considered for inclusion in the MMI system. The final survey for the MMI system consisted of 32 measures and demographic items (Table 3).

**Table 3.** Measures included in the multiscale modeling and intervention (MMI) system.

Construct	Measure	Description	Psychometric properties
Overall mental and physical health	PROMIS^a^ Global Health	A 10-item measure of a person’s overall evaluation of their health	The scale has evidence for construct validity and adequate internal consistency (Global Physical Health Cronbach *α*=0.81; Global Mental Health Cronbach *α*=0.86) [21].
Medical comorbidities	Self-Administered Comorbidity Measure	An instrument that assesses for medical comorbidities and functions	The instrument has evidence for construct validity and adequate test-retest reliability (intraclass correlation coefficient=0.94, Pearson *r*=0.81) [22].
Side effects and symptoms associated with endocrine therapy	Breast Cancer Prevention Trial Checklist	A 42-item measure of vasomotor symptoms, urinary incontinence, cognitive and mood symptoms, vaginal symptoms, and weight gain/appearance concerns	The subscales have evidence for construct validity and adequate internal consistency (Cronbach *α*>0.70) [23,24].
Sleep-related impairment	Sleep-Related Impairment Short Form 8a	An 8-item measure of perceptions of sleep alertness, sleepiness, and tiredness during usual waking hours, and the perceived functional impairments during wakefulness	The scale has evidence for construct validity and adequate internal consistency (Cronbach *α*=0.91) [25].
Sleep disturbance	Sleep Disturbance—Short Form 8b	An 8-item measure of perceptions of sleep quality, sleep depth, and restoration associated with sleep	The scale has evidence for construct validity and adequate internal consistency (Cronbach *α*=0.84) [25].
Fatigue	Fatigue Symptom Inventory	A 13-item measure of multiple aspects of fatigue, including severity, frequency, and interference with daily functioning	The scale has evidence for construct validity and adequate internal consistency (Cronbach *α*=0.94) [26,27].
Region of pain	Pain Region Scale	A measure of degree of bother for pain in the stomach, head, neck or back, widespread, and in the arms, legs, or joints	Psychometric evidence is not available because the Pain Region scale was developed by the research team.
Pain intensity	PROMIS Pain Intensity 3 a Short Form	A 3-item measure of degree to which a person hurts	The scale has evidence for construct validity and adequate internal consistency (Cronbach *α*=0.86) [28].
Catastrophic pain−related thoughts	Pain Catastrophizing Scale	A 13-item measure of catastrophic pain-related thoughts, including rumination, magnification, and helplessness	The scale has evidence for construct validity and adequate internal consistency (Cronbach *α*=0.75-0.93) [29].
Hot flash interference	Hot flash−Related Daily Interference Scale	A 3-item measure of perceived hot flash interference, referring to the degree to which hot flashes interfere with 9 aspects of daily life and overall quality of life.	The scale has evidence for construct validity and adequate internal consistency (Cronbach *α*>0.90) [30].
Cognitive function	PROMIS Cognitive Function-Short Form 8a	An 8-item measure of perceived cognitive deficits	The scale has evidence for construct validity and adequate internal consistency (Cronbach *α*=0.89-0.97) [31].
Personality traits	Mini IPIP^b^	A 20-item measure of the following 5 personality dimensions: extraversion, agreeableness, conscientiousness, neuroticism, and intellect/imagination	The subscales have evidence for construct validity and adequate internal consistency (Cronbach *α*>0.70) [32].
Perceived control	Pearlin Mastery Scale	A 7-item measure of perceptions of control or the extent to which one regards one’s life chances as being under one’s own control in contrast to being fatalistically ruled	The scale has evidence for construct validity and adequate internal consistency (Cronbach *α*>0.70) [33].
Spirituality	The Functional Assessment of Chronic Illness Therapy-Spiritual Well-Being	A 12-item measure of spirituality of patients with chronic and/or life-threatening illness	The scale has evidence for construct validity and adequate internal consistency (Cronbach *α*>0.80) [34].
Perceived stress	The Perceived Stress Scale	A 10-item measure of the degree to which individuals feel that situations in their lives are unpredictable, uncontrollable, and overloading.	The scale has evidence for construct validity and adequate internal consistency (Cronbach *α*>0.80) [35].
Generalized anxiety	Generalized Anxiety Disorder Scale	A 7-item measure of anxiety symptoms within the previous 2 weeks	The scale has evidence for construct validity and adequate internal consistency (Cronbach *α*>.80) [36].
Depression	Patient Health Questionnaire Depression Scale	An 8-item measure of depressive symptoms within the previous 2 weeks	The scale has evidence for construct validity and adequate internal consistency (Cronbach *α*=0.86) [37,38].
Fear of cancer recurrence	Fear of Cancer Recurrence Inventory-Short Form	A 9-item measure of fear of cancer recurrence, referring to the fear or worry that the cancer will return or progress in the same organ or in a different part of the body	The full scale has evidence for construct validity and adequate internal consistency (Cronbach *α*>0.71) [39].
Adherence to endocrine therapy	The Medication Adherence Report Scale	A 5-item measure of adherence with endocrine therapy	The scale has some evidence for construct validity and adequate internal consistency (Cronbach *α* range=0.67-0.89) [40].
Self-efficacy in adherence to medications	Medication Adherence Self-Efficacy Scale	A 26-item measure of self-efficacy in adherence to prescribed medications	The original scale has evidence for construct validity and adequate internal consistency (Cronbach *α*=0.95) [41].
Beliefs about medicines	The Beliefs about Medicines Questionnaire	An 18-item measure of commonly held beliefs about medicines	The scale has evidence for construct validity and adequate internal consistency (Cronbach *α*>0.70) [42,43].
Perceived necessity of endocrine therapy	Perceptions of endocrine therapy necessity items	Two items are used to measure perceived necessity of endocrine therapy	Psychometric evidence is not available on the items, but they have been used in previous cancer-related studies.
Decision regret	The Decision Regret Scale	A 5-item measure of distress or remorse after choosing to take or not take endocrine therapy	The scale has evidence for construct validity and adequate internal consistency (Cronbach *α*>0.70) [44].
Satisfaction with cancer health care	Patient Satisfaction with Cancer Care	An 18-item measure of perceived satisfaction with health care received since diagnosis of cancer	The measure has evidence for construct validity and high internal consistency (Cronbach *α* range=0.95‐0.96) [45-47].
Emotional support	PROMIS Emotional Support	An 8-item measure of perceived feelings of being cared for and valued as a person, having confident relationships	The scale has adequate construct validity and internal consistency (Cronbach *α*=0.97) [48].
Instrumental support	PROMIS Instrumental Support	An 8-item measure of perceived availability of assistance with material, cognitive, or task performance	The scale has evidence for construct validity and adequate internal consistency (Cronbach *α*=0.95) [48].
Informational support	PROMIS Informational Support	A 10-item measure of perceived availability of helpful information or advice	The scale has evidence for construct validity and adequate internal consistency (Cronbach *α*=0.96) [48].
Barriers to receiving care	The Barriers to Care Scale	A 28-item measure of barriers to health care related to geography/distance, medical and psychological issues, community stigma, and personal resources	The original scale has evidence for construct validity and adequate internal consistency (Cronbach *α*>0.70) [49].
Cancer self-efficacy	The Communication and Attitudinal Self-Efficacy	A 12-item measure of self-efficacy in dealing with cancer and related health services	The measure has evidence for construct validity and adequate internal consistency (Cronbach *α*>0.80) [50].
Participation in health care decisions and quality of relationship with health care provider	Participation in Decision and Relationship Quality Survey	A 9-item measure of support received from doctors and other health professionals, the patient’s role in decision-making, information shared with the patient about potential side effects, and their experiences with feeling listened to and respected by their provider	Psychometric evidence is not available on the items, but they have been used in previous cancer-related studies.
Social-psychological aspects of patient-physician interactions	Interpersonal Processes of Care Survey: Short Form	An 18-item measure of the social-psychological aspects of the patient-physician interaction	The scale has evidence for construct validity and adequate internal consistency (Cronbach *α*>0.70) [51].
Self-efficacy when interacting with physicians	Patient-Perceived Self-Efficacy	A 10-item measure of subjective sense of patients’ confidence when interacting with their physician	The scale has evidence for construct validity and adequate internal consistency (Cronbach *α*>0.90) [52].

a PROMIS: patient-reported outcomes measurement information system.

b Mini IPIP: Mini International Personality Item Pool.

#### Summary of the MMI System

The sensors selected for the final MMI system include an RxCap smart pill bottle and its associated smartphone app, the Fitbit Sense smartwatch and its associated smartphone app, and the Sensus Mobile smartphone app, which includes smartphone sensors and can administer EMA surveys. The timing of the EMA surveys includes morning and evening surveys, randomized surveys, surveys administered every 2 weeks, and surveys administered every 4 weeks. Additionally, the study team identified 32 constructs that will be included in the long-form baseline, 3-month, and 6-month surveys for the MMI system.

## Discussion

### Principal Findings

The purpose of this study was to describe the development of the MMI system, a novel approach to improving adherence to ET among early-stage breast cancer survivors that aims to understand medication adherence barriers and medication-taking behaviors. Overall usability testing interview findings suggest that participants were willing to use each component of the entire MMI system for the future 6-month-long study duration. A secondary analysis of data identified 104 survey items with significant contributions to 4-week medication adherence. Forty-two additional surveys were reviewed as predictors of ET medication adherence, and as per the SCT and other health and communication theories, included personal factors, environmental factors, and the behavior itself. When combined, the RNN analysis [19] and the review of existing literature resulted in the identification of 34 surveys that could be included in the MMI system during the planned deployment, measuring constructs identified in both approaches as important predictors of ET adherence.

### Comparison to Prior Work

While interventions that aimed to improve ET adherence have been found to be effective overall, there is significant heterogeneity in the effects of psychosocial interventions, with reminder interventions being the most consistently helpful [13]. The lack of effectiveness of some psychosocial ET adherence intervention approaches suggests a lack of understanding of the barriers to ET adherence and the context of medication-taking behavior. The MMI system is designed to overcome challenges in improving adherence to daily ET among breast cancer survivors in stages 0 to 3 who have completed surgery, chemotherapy, and radiation. Personalized approaches to improving medication-taking behaviors may address the varied efficacy of prior psychosocial interventions to increase ET adherence among breast cancer survivors [13] by understanding personal, environmental, and behavioral factors as described in the SCT, along with understanding reciprocal determinism from the SCT, that is, the fact that all of the factors (including the behavior and medication adherence) influence each other in a dynamic way.

### Strengths and Limitations

The strengths of the current work are that we used a novel and comprehensive approach to creating an MMI system through RNN analysis findings, usability interviews, and a review of research literature of constructs and the measurement of constructs associated with ET adherence among breast cancer survivors. Additionally, our research highlights the use of theory, data-driven models, and participant feedback to inform intervention development. This is not without limitations. First, the breast cancer survivors recruited during the usability interviews, which occurred during the COVID-19 pandemic lockdowns, were primarily White. Thus, the usability interview findings may not generalize to other ethnic/racial groups. However, the secondary analysis participants were diverse, and the study team will focus on recruiting more diverse participants for phase 2 of the study (see Figure 1) to mitigate this limitation. Second, participants were primarily recruited via social media posts, so they may be inherently different, such as potentially being more familiar with and/or eager to adopt technologies included in the MMI system, compared to the general population of breast cancer survivors. To mitigate this limitation in the phase 2 deployment, the study team has increased recruitment strategies to include breast cancer survivors from various sources (ie, ResearchMatch and in-person recruitment). Third, the secondary data analysis was conducted on diverse female breast cancer survivors as part of a different intervention study; therefore, the original survey development was not intended to be used for the analytic purposes used in this study. Therefore, we were unable to include newly identified modifiable factors in the secondary data analysis. However, the purpose of the RNN was to develop and test the analytical framework that provides critical insight and refinement into the desired models to be run in phases 2 and 3 (Figure 1) of the project. Fourth, the analysis of the interview transcripts was completed by a single reviewer. Future research may benefit from incorporating additional reviewers into the analysis of the transcripts to better confirm the findings.

### Future Directions

The next phase of the project (phase 2) will include the deployment of the entire MMI system with 20 breast cancer survivors over 6 months, collecting data on constructs from multiple health-related theories. Using the data collected during the planned phase 2 deployment, the RNN analysis will be used to identify the most important factors that contribute to ET nonadherence at various time frames among a new sample of breast cancer survivors, including more dynamic features, such as medication-taking patterns, data collected from wrist-worn sensors, smartphone sensors, and EMA. In subsequent deployment (phases 2 and 3, see Figure 1), we will continue to collect information on perceptions of devices, systems, and surveys in the MMI system to better refine it. The study team will then work toward reducing the amount of data collected to essential surveys, EMA, and sensors. Furthermore, the study team will begin to develop intervention components based on the group analysis and individualized model analyses in preparation for phase 3, which is the deployment of the refined MMI system and the implementation of the individualized intervention components that deliver intervention content based on a person’s particular risks for ET nonadherence across different time frames (eg, daily and weekly). These intervention strategies may draw from multiple health and communication theories and can be matched to app features (eg, information reminders, reinforcement, and praise), which will likely improve medication adherence across different timescales [53]. Future large-scale testing of the MMI system and intervention approaches will be required to determine its effectiveness in increasing ET adherence among breast cancer survivors.

## Supplementary material

10.2196/68255 Multimedia Appendix 1 Characteristics of wearable sensor and medication event monitoring system devices reviewed.

10.2196/68255 Multimedia Appendix 2 Interview guides.

10.2196/68255 Checklist 1 COREQ checklist.

## Abbreviations

COREQ: Consolidated Criteria for Reporting Qualitative Research
EMA: ecological momentary assessment
ET: endocrine therapy
IRB: institutional review board
MEMS: medication event monitoring system
MMI: multiscale modeling intervention
RCT: randomized controlled trial
RNN: randomized neural network
SCT: social cognitive theory
