# Supplementary material for: Improving Long-Term Adherence to Endocrine Therapy Among Breast Cancer Survivors: Development of a Multiscale Modeling and Intervention System
Source: JMIR Cancer. 2026 Apr 30;12:e68255. doi: 10.2196/68255 (PMC13131827; doi:10.2196/68255)
Supplement: Multimedia Appendix 1 [file cancer-v12-e68255-s001.pdf]

S1. Characteristics of wearable sensor devices tested.

	Device	Worn	Platform(s)	Connectivity	Cost in US Dollars	Battery Life (all sensors on)	Limitations
**Apple Watch**							
		Wrist	iOS	Bluetooth, Wifi, LTE	$499	1 hour	Do not support continuous heart rate variability data. Price is an issue.
**Oura Ring**							
		Finger	No OS	Bluetooth	$299	Less than 1 hour	
**Huawei Watch**							
		Wrist	Lite OS	Bluetooth, Wifi	$250	1.5 hours	Lite OS is less supportive
**Samsung Gear S3**							
		Wrist	Tizen	Bluetooth, Wifi	$299	10 hours (GPS mode with streaming music)	Tizen is less supportive. Also several issues regarding battery drain were reported.
**Garmin Flex 6**							
		Wrist	Garmin Watch OS	Bluetooth, Wifi	$699	16 hours (GPS mode with streaming music)	RR interval data available. Price is an issue.
**Fitbit Ionic**							
		Wrist	Fitbit OS	Bluetooth, Wifi	$250	10 hours (GPS on)	No raw data available.

S2. Characteristics of medication event monitoring system devices reviewed.

	Device	Format	Platform(s)	Connectivity	Feedback	Cost in US Dollars

**Med-ic intelligent blister package**						
		Blister	Android, iOS	WiFi	Date and time each unit dose is removed	Unspecified
**MedsOnTime (many options)**						
		Box	Android, iOS	Unspecified	Sound / light reminders to take medication, and warnings about double doses	$39.90 upwards (one-time payment)
**Pill drill**						
		Box	Unspecified	Unspecified	Unspecified	$279
**SimpleMed+**						
		Box	Unspecified	Bluetooth, GSM (cellular)	Reminders, blinking light	Unspecified
**Elliegrid**						
		Box	Android, iOS	Bluetooth	Reminders, solid lights	$149
**Pillbox by Tricella**						
		Box	Android, iOS	Bluetooth	Unspecified	$94.99
**MedMinder Smart Automated Pill Dispenser**						
		Box	Unspecified	GSM (cellular)	Reminders, blinking light, beeping	$49.99 per month
**(Aardex) MEMS Cerepak**						

		Blister	Android, iOS	USB, Other	Compliance and dosing behaviors, reminders	Unspecified
**(Aardex) MEMS Cap**						
		Cap	Android, iOS	USB, Other	Reminders, compliance and dosing behaviors	Unspecified
**Pillsy**						
		Cap	Android, iOS	Bluetooth	Reminders, flashing light	$44.95 per large container
**eCAP**						
		Cap	Android, iOS	Unspecified	Unspecified	Unspecified
**adheretech**						
		Cap	Unspecified	Unspecified	Reminders	Unspecified
**Medikyu**						
		Cap	Android, iOS	Bluetooth	Alarm	$59.99 for an orange or white bottle
**RxCap**						
		Cap	Android, iOS	Bluetooth	Reminders, flashing light, beeping	$39.99 one-time payment
**(Aardex) MEMS Electronic Dosepak**						
		Blister	Android, iOS	USB, Other	Reminders, compliance and dosing behaviors	Unspecified
**(Aardex) MEMS Helping Hand**						
		Blister	Android, iOS	USB, Other	Reminders, compliance and dosing behaviors	Unspecified

**Intent Solutions TAD**						
		Box	Unspecified	Bluetooth	Unspecified	Unspecified
**(Aardex) MEMS Button**						
		Box	Android, iOS	USB, Other	Reminders, compliance and dosing behaviors	Unspecified
