# Supplementary material for: Improving Long-Term Adherence to Endocrine Therapy Among Breast Cancer Survivors: Development of a Multiscale Modeling and Intervention System
Source: JMIR Cancer. 2026 Apr 30;12:e68255. doi: 10.2196/68255 (PMC13131827; doi:10.2196/68255)
Supplement: Multimedia Appendix 2 [file cancer-v12-e68255-s002.docx]

**ROUND 1 INTERVIEW GUIDE**

1. In general, how did you feel about the experience of filling out the surveys on the smartphone?

1. What did you like most about using this smartphone app?

1. What did you like least about using this smartphone app?

1. How easy or difficult was it to answer the questions in the app?

1. How would you feel if we were to ask you to use this smartphone app for a long

period of time, like 6 months?

1. Would you find it easy or difficult to use the smartphone app on your own phone?

1. How often would you be able to fill out surveys like this on your smartphone?

1. About how many questions like this would you be able to fill out at a time?

1. Are there times of the day where it would be easier for you to answer survey

questions like these?

1. Are there times of the day where it would be harder for you to answer survey

questions like these?

1. Are there times of the day where it would be impossible for you to answer survey

questions like these?

1. Are there days of the week where it would be easier for you to answer survey

questions like these on these days?

1. Are there days of the week where it would be harder for you to answer survey

questions like these on these days?

1. Are there days of the week where it would be impossible for you to answer

survey questions like these?

1. How do you feel about the notifications from the app?

1. Do you have other thoughts that you would like to share about the smartphone app?

**ROUND 2 INTERVIEW GUIDE**

1. What do you think about the size of the smartwatch?

1. What do you think about the appearance of the smartwatch?
2. What do you think about these smartwatch options?

**FOR PARTICIPANTS WHO DO NOT HAVE A SMARTWATCH:**

4. How would you feel if we were to ask you to wear a smartwatch every day for a long period of time, like 6 months?

5. Would you find it easy or difficult to remember to wear the smartwatch every day?

6. Would you find it difficult or easy to remember to charge the smartwatch at night?

7. Tell me about any times when you would not want to wear the smartwatch?  Why wouldn’t you want to wear it then?

**FOR PARTICIPANTS WHO ALREADY HAVE A SMARTWATCH:**

8. How would you feel if we were to ask you to wear a smartwatch for our project every day for a long period of time, like 6 months? This would be a different smartwatch from the one that you already have.

9. Would you find it easy or difficult to remember to wear the project smartwatch every day?

10. Would you find it difficult or easy to remember to charge the project smartwatch at night?

11. Tell me about any times when you would not want to wear the project smartwatch?

**FOR ALL PARTICIPANTS:**

12. In general, how did you feel about the experience of answering the surveys on the smartwatch?

13. What did you like most about answering surveys on the smartwatch?

14. What did you like least about answering surveys on the smartwatch?

15. How easy or difficult was it to answer the survey questions?

16. What do you think about the appearance of the smartwatch app?

17. How would you feel if we were to ask you to answer questions like these on the

smartwatch that we gave you for a long period of time, like 6 months?

18. Would you find it easy or difficult to use the survey smartwatch app on a

smartwatch we gave you?

19. How often would you be able to fill out surveys like this on a smartwatch we gave

you?

20. About how many questions like this would you be able to fill out at a time?

21. Are there times of the day where it would be easier for you to answer survey

questions like these on the smartwatch?

22. Are there times of the day where it would be harder for you to answer survey

questions like these on the smartwatch?

23. Are there times of the day where it would be impossible for you to answer survey

questions like these on the smartwatch?

24. Are there days of the week where it would be easier for you to answer survey

questions like these on the smartwatch?

25. Are there days of the week where it would be harder for you to answer survey

questions like these on the smartwatch?

26. Are there days of the week where it would be impossible for you to answer

survey questions like these on the smartwatch?

27. How do you feel about the notifications from the app?

28. Do you have any other thoughts that you would like to share about answering

surveys like this on your smartwatch?

**ROUND 3 INTERVIEW GUIDE**

1. How many prescription medicines do you currently take each day?

1. How many vitamins or supplements do you currently take each day?

1. What times of the day do you take your medicines and supplements?

1. Where do you keep your medicines and supplements (e.g., purse, night stand,

medicine cabinet)?

1. In what type of container do you keep your medicines and supplements? (e.g., in

a pill keeper, in the original bottle)?

1. Do you have any medicines that come in a blister pack or some other type of

dispenser?

1. Do you have any medicines that are liquid?

1. Do you have any medicines that need to be refrigerated?

1. Do you sometimes have a different container for your medicines and

supplements, like a smaller container that you take on a trip or an outing?

1. Does anyone help you with managing or taking your medicines?

1. Have you ever seen a smart pill bottle or box like this?

1. Have you ever used a smart pill bottle or box like this?

1. What did/do you think about the smart pill bottle or box that you used?

1. Do you have a smartphone?

1. Have you ever used an app on your phone to manage your medicines?

1. What do you/did you think about the app that you use to track your medicines?

17. What did you think about the smart pill bottle?

18. What do you like most about the smart pill bottle?

19. What do you like least about the smart pill bottle?

20. How easy or difficult do you think it would be to open this smart pill bottle?

21. How easy or difficult do you think it would be to use the smart pill bottle in your

everyday life?

22. If you could change something about the smart pill bottle, what would it be?

23. How would you feel if we were to ask you to use this smart pill bottle for a long

period of time, like 6 months?

24. Would it be easy or difficult to fit using this smart pill bottle into your everyday life?

25. Would you be able to still use this pill bottle if you take multiple medicines each day?

26. How would you handle using this smart pill bottle if you had to be away from the place where you usually take your medicines, like if you had to take a trip away from home?

27. How do you think using this smart pill bottle would affect the routine that you have for

taking your medicines?

28. Do you think that using this smart pill bottle for a long time would make it more or less likely that you would forget your medicines?

29. Do you think that it would be easy or difficult to keep track of this smart pill bottle?

30. Do you have other thoughts that you would like to share about the smart pill bottle?

**ROUND 4 INTERVIEW GUIDE**

1. How many prescription medicines do you currently take each day?

1. How many vitamins or supplements do you currently take each day?

1. What times of the day do you take your medicines and supplements?

1. Where do you keep your medicines and supplements (e.g., purse, night stand,

medicine cabinet)?

1. In what type of container do you keep your medicines and supplements? (e.g., in

a pill keeper, in the original bottle)?

1. Do you have any medicines that come in a blister pack or some other type of

dispenser?

1. Do you have any medicines that are liquid?

1. Do you have any medicines that need to be refrigerated?

1. Do you sometimes have a different container for your medicines and

supplements, like a smaller container that you take on a trip or an outing?

1. Does anyone help you with managing or taking your medicines?

1. Have you ever seen a smart pill bottle or box like this?

1. Have you ever used a smart pill bottle or box like this?

1. What did/do you think about the smart pill bottle or box that you used?

1. Do you have a smartphone?

1. Have you ever used an app on your phone to manage your medicines?

1. What do you/did you think about the app that you use to track your medicines?

17. What did you think about the smart pill box?

18. What do you like most about the smart pill box?

19. What do you like least about the smart pill box?

20. How easy or difficult do you think it would be to open this smart pill box?

21. How easy or difficult do you think it would be to use the smart pill box in your everyday life?

22. If you could change something about the smart pill box, what would it be?

23. How would you feel if we were to ask you to use this smart pill box for a long period of time, like 6 months?

24. Would it be easy or difficult to fit using this smart pill box into your everyday life?

25. Would you be able to still use this pill box if you take only one pill each day?

26. How would you handle using this smart pill box if you had to be away from the place where you usually take your medicines, like if you had to take a trip away from home?

27. How do you think using this smart pill box would affect the routine that you have for taking your medicines?

28. Do you think that using this smart pill box for a long time would make it more or less likely that you would forget your medicines?

29. Do you think that it would be easy or difficult to keep track of this smart pill box?

30. Do you have other thoughts that you would like to share about the smart pill box?

**ROUND 5 INTERVIEW GUIDE**

1. What are your overall thoughts on the survey?

1. What did you like about the survey?

1. What didn’t you like about the survey?

1. Were there any questions that you found confusing?

1. Were there any questions that were upsetting?

1. What are your overall thoughts on the Fitbit?

1. What are your thoughts on asking people to wear the Fitbit daily for six months?

1. What do you think about asking people to charge the Fitbit every two to three

days?

1. What are your overall thoughts on the RxCap?
2. How likely do you think you could use the RxCap for six months with your

endocrine therapy medicine?

1. What are your overall thoughts on the Sensus app?

1. What did you like about the Sensus app?

1. What didn’t you like about the Sensus app?

1. How would you feel about answering questions every few days on Sensus?

1. Describe your comfort level with having Sensus collect these types of data from

your phone.

1. What do you think about having options to let the Sensus app collect information

or not collect it?

1. Would you participate in a study where you had to use the entire system for 6

months and you received incentives for using it (but not any assistance with taking your medicine)?

1. Would you use this system if it helped you with taking your medicines?

1. Do you have other thoughts or questions that you would like to share about the

system or specific components?
